# Prevalence of hepatitis B and C viral co-infection and associated factors with HIV infection in children in South Kivu, Democratic Republic of the Congo

**DOI:** 10.1186/s12879-023-08474-8

**Published:** 2023-08-14

**Authors:** Serge Ahuka Miyanga, Tony Akilimali Shindano, Etienne Mwamba Shindano, Célestin Bisangamo Kyambikwa, Jeff Maotela Kabinda

**Affiliations:** 1 Department of Paediatrics, Hôpital Provincial Général de Référence de Bukavu (HPGRB), Bukavu, DR Congo; 2 Department of Internal Medicine, Hôpital Provincial Général de Référence de Bukavu (HPGRB), Bukavu, DR Congo; 3 Faculty of Medicine, Université Catholique de Bukavu (UCB), PB 285, Bukavu, South Kivu Democratic Republic of the Congo; 4 University of Kindu, Democratic Republic of the Congo, Kindu, Maniema, Democratic Republic of the Congo; 5 Institut Supérieur des Techniques Médicales de Bukavu, Sud Kivu, DR Congo; 6 Université Pédagogique Nationale, Kinshasa, DR Congo

**Keywords:** HIV, HBV, HCV, Co-infection, Children, South Kivu

## Abstract

**Background:**

The World Health Organization’s (WHO) 2030 goal of eradicating Hepatitis B and C viruses must also include HIV co-infected children. However, data on the prevalence of this condition are lacking in the Democratic Republic of Congo (DRC), which is considered as one of the countries with high-prevalence of these viruses. The need to assess the extent of this co-infection in the children of this country is therefore important in order to capitalize on efforts to improve prevention and management of both infections.

**Methodology:**

This is a comparative cross-sectional study conducted from February 04, 2015 to September 03, 2019 at 14 General Reference Hospitals with a pediatric HIV management programme in South Kivu province. The study compared the frequency of hepatitis B (HBV) and C (HCV) markers and factors associated with these two viruses in two equal groups: HIV-positive and HIV-negative children. The data were analyzed using the SPSS version 20.0 software and the significance level was set at p-value less than 0.05.

**Results:**

The study involved a total of 594 children, 297 of whom were HIV-positive and 297 negative. HBsAg was found in 8.7% of HIV-positive patients and 0.7% for HCV antibodies. On the other hand, among the HIV-negative patients, the proportion of HBsAg was 0.7% but no cases with anti-HCV antibodies were detected. HIV status increases by 14 times the risk of co-occurring with HBV [OR 14.1 (95% CI: 3.33–60.2); p < 0.001] and this risk is not apparent for HCV (p = 0.297). Multivariate logistic regression showed that history of jaundice in the family (aOR:4.19;95% CI: 2.12–11.59), recent hospitalization (aOR:10.7;95% CI: 6.69–17.2), surgery (aOR: 3.24;95% CI: 1.18–8.92), piercing (aOR: 4.26;95% CI: 1.70–10.7) and transfusion in the last 6 months (aOR: 2.69;95% CI: 1.55–4.67) were significantly associated with higher risk of being HBV- HIV co-infected.

**Conclusion:**

This study investigated the importance of hepatitis viral co-infections in HIV-positive children in South Kivu. Particular attention should be paid to prevention and early detection of these co-infections in this population.

## Introduction

Chronic Human Immunodeficiency Virus (HIV), hepatitis B (HBV) and hepatitis C (HCV) infections are currently three of the most documented chronic viral infections in the world [1, 2]. In 2019, the World Health Organization (WHO) reported that approximately 38 million (31.6–44.5 million) people were living with HIV, 2/3 of them in Asia and Africa [3, 4]. On the other hand, approximately 257 million were chronic carriers of HBV out of 2.5 billion individuals who had contracted this virus and 71 million had chronic HCV infection [4]. Given the similarity of their risk factors and routes of transmission, it is estimated that of these 38 million people living with HIV, 2.7 million would also be at the same time affected by HBV and 2.3 million by HCV [3]. HBV/HCV and HIV co-infections have therefore become a real public health problem on a global scale, more particularly in areas of high endemicity for these three viruses. This is because they have become leading factors of comorbidity and mortality apart from HIV, due to the increase in the lifespan of treated patients [5]. These three viruses also share the same modes of transmission in children. For HIV, transmission is essentially vertical and from blood products, but the generalization of short regimens of antiretrovirals from the third trimester of pregnancy could help to reduce the transmission rates below 5% [6]. For HBV, apart from transmission by blood products [7, 8], mother-to-child (MTC) transmission remains the common way of dissemination in highly-endemic countries, especially when the mother is HBeAg positive, as in Sub-Saharan Africa [8]. This rate of MTC transmission of HBV is estimated to be between 40 and 45% [9]. For HCV, transmission in children occurs almost exclusively by parenteral route in Africa while the risk of MTC transmission remains low around 5% [10]. In HIV-infected children, testing for HBV and HCV is, therefore, important, if not essential, in order to prevent complications related to the acceleration of their expression on the function of the liver, compromising at the same time the response to antiretrovirals and reducing their survival [11]. This is especially important considering that early transmissions of viral hepatitis during first years of life are associated with more chronic disease and fatal complications.

The Democratic Republic of Congo (DRC) is considered as one of the countries with high-prevalence of these three viruses but the extent of HIV/Hepatitis B and C co-infection in children is poorly documented. In this context, the risk of MTC transmission should be important because of the high prevalence of these two viruses in pregnant and lactating women. Previous data in pregnant women showed prevalence rates around 5% and 3% for hepatitis B and C, respectively [12–14].

It is in this context that the DRC has implemented a universal infant HBV immunization programme consisting of administration of three doses of this vaccine at the ages of 6, 10 and 14 weeks. However, during the study period, national surveys showed that the hepatitis B vaccination coverage varied between 45 and 60%. At the same time, around 65% of 1 to 3 month old children were exclusively breastfed [15].

In DRC, the HIV prevalence hovers around 1% among adults but data on specific rate in children are limited [16, 17]. Knowledge of the profile of co-infections by these three viruses is a key issue in the control of these diseases potentially transmitted from mother to child, especially within the global HIV/HBV elimination project.

This study determined the seroprevalence and associated factors of HBV and HCV co-infections in HIV-positive children.

## Methodology

### Type and setting of study

This was a comparative cross-sectional study in which patients were recruited in 14 General Reference Hospitals organizing a HIV management program for children in the Province of South Kivu (eastern DRC). These hospitals care for both HIV and non-HIV patients. Six of them are located in urban areas and eight are in rural areas.

### Study population and sampling

The target population consisted of 2 to 16-year-old children, who have been diagnosed with HIV infection and are cared for. This group of patients was compared to another group with the same size made up of children recruited in the same hospitals sharing similar characteristics, except for the HIV status. In these two groups, participants were continuously enrolled until achieving the necessary sample size and they were included in the respective groups according to their HIV status.

The minimum sample size was determined using the following formula for calculating sample size for the comparison of two independents proportions: [18]

1 $$n{\text{ }}per{\text{ }}group = \frac{{2{{({Z_\alpha } + {Z_\beta })}^2}p(1 - P)}}{{{{(Po - {p_1})}^2}}}$$

Where.

- Zα = standard normal deviate of α at 95% confidence interval (type 1 error) = 1.96.
- Zβ = standard normal deviate of β (the power), fixed at 90% = 1.28.
- P_0_ is the proportion of HBV co-infection in HIV children. A prevalence of 13.5% was used based on the work by Kitetele et al. [19].
- P_1_ is the proportion of hepatitis B in children. A prevalence of 3.6% was found by Kabinda et al. [20]
- P is the arithmetic average of these two previous proportions : 1/2 (P_0_ + P_1_).

The calculated minimal sample size per group was 167 children but, in order to boost the power of statistical tests, we decided to increase the size by more than 50% in each group. At the end, we were able to recruit 297 patients per group.

### Data collection and laboratory analysis

- The general characteristics (socio-demographic data) as well as medical information of children and their mothers (medical history of HIV infection and possible risk factors for hepatitis transmission) were collected using a survey questionnaire and data from medical records.
- Sample collection and biomedical analyses: 2 to 3 milliliters of whole blood were collected and placed in two EDTA tubes. Before plasma separation, two blood spots were then collected and placed onto Whatman 903 ® filter paper to enable the realization of anti-HIV serological tests (Determine® and ELISA brand Dialab®HIV Ab Sensitive, Eastleigh, UK), the search for HBsAg of HBV (Determine® and ELISA Dialab® HBsAg Sensitive, Eastleigh, UK) and anti-HCV antibodies (Dialab®HCV Ab Sensitive ELISA Kit, Eastleigh, UK). The sensitivity and specificity of all tests were over 98 and 99%. The results were interpreted according to the manufacturer’s specifications.

### Statistical analyses

The collected data were analyzed using the SPSS version 20.0 software (Chicago, SPSS Inc.). Qualitative variables are represented as numbers and percentages, while for continuous quantitative variables, their median (percentiles 25–75) has been determined. The normality of the distribution was verified by the Kolmogorov-Smirnov test. The search for possible associations between the dependent variables and the independent variables, the Pearson chi-square test (or the exact Fischer test) was used according to the conditions of application. Odds ratio with 95% confidence interval were calculated at a significance level of p < 0.05. All explanatory variables with a p value ≤ 0.2 in the bivariate analysis were included in the multivariate logistic regression model to identify independent predictive variables.

### Ethical considerations

The study obtained the authorization of the ethics committee of the Catholic University of Bukavu (UCB/CIES/NC/030/2021). The anonymity of children and their mothers was assured. In addition, the children’s parent or guardian signed an informed consent form before involvement into the study. Children with positive HBsAg or anti-HCV antibodies were referred to a hepatologist for further investigation and management.

## Results

The study analyzed data of 594 children and their mothers, including 297 by subgroup. The analysis of general characteristics did not show any statistical difference between the two groups in relation to socio-demographic data of children and their parents (Table 1).

**Table 1:** Characteristics of children and mothers according to child HIV status

General features	Total n (%)	HIV positive n (%)	HIV negative n (%)	p-value
Child’s age in months				
Median age (P25-P75)	89.0 (74.0-104)	94.0 (73.0-115)	86.0 (72.0-100)	0.121
24–59	206 (33.7)	98 (33.0)	118 (39.7)	0.088
≥ 60	388 (65.3)	199 (67.0)	179 (60.3)	
Child’s gender				
Female	269 (45.3)	125 (42.1)	144 (48.5)	0.117
Male	325 (54.7)	172 (57.9)	153 (51.5)	
Mother’s age in years				
< 18	9 (1.5)	7 (2.4)	2 (0.7)	0.068
18–35	255 (42.9)	136 (45.8)	119 (40.1)	
≥ 36	330 (55.6)	154 (51.8)	176 (59.2)	
Mother’s marital status				
Single	13 (2.2)	8 (2.7)	5 (1.7)	0.646
Married	37 (6.2)	17 (5.7)	20 (6.7)	
Separated or divorced	387 (65.1)	189 (63.6)	198 (66.7)	
Widowed	157 (26.5)	83 (28.0)	74 (24.9)	
Mother’s level of education				
Without or primary	283 (47.6)	144 (48.5)	139 (46.8)	0.681
High school or university	311 (52.4)	153 (51.5)	158 (53.2)	
Mother’s occupation				
Without	60 (10.1)	36 (12.1)	24 (8.1)	0.012
Liberal	288 (48.5)	155 (51.2)	133 (44.8)	
State official	246 (41.4)	106 (36.7)	140 (47.1)	
Maternal HIV serology				
Known	490 (82.5)	251 (84.5)	239 (80.5)	0.195
Unknown	282 (17.5)	26 (15.5)	256 (19.5)	
Father’s HIV serology				
Known	352 (59.3)	184 (62.0)	168 (56.6)	0.181
Unknown	242 (40.7)	107 (38.0)	129 (43.4)	
Place of provenance				
Rural	241 (40.6)	119 (40.1)	122 (41.1)	0.802
Urban	353 (59.4)	178 (59.9)	175 (58.9)	

Viral hepatitis B and C infections were significantly higher in HIV + patients than in seronegative patients (Tables 2 and 3). However, only HBV showed a statistically significant association with HIV infection. The proportions of HBV in HIV-positive and HIV-negative children were 8.7% and 0.7% (p < 0.001), respectively, while those of HCV were 0.7% and 0.0% (p = 0.297). Seroprevalence remains higher in all age groups of HIV + patients while no cases of hepatitis B using HBsAg test have been observed in HIV-negative children under 60 months of age. In general, HIV status multiplies the risk of presenting with HBV concomitantly by 14 times (95% CI: 3.33–60.2). Table 4 presents the results of the multivariate analysis for factors potentially associated with HIV-viral hepatitis B co-infection. Compared to the analysis of potential risk factors for HBV in HIV positive patients, some factors stand out clearly, in particular the fact of having been transfused, tattooed, and pierced or a family history of jaundice. Analysis after controlling for possible confounders showed that history of recent hospitalization (aOR:10.7;95% CI: 6.69–17.2), jaundice in the family (aOR:4.19;95% CI: 2.12–11.59), surgery (aOR: 3.24;95% CI: 1.18–8.92), piercing (aOR: 4.26;95% CI: 1.70–10.7) and transfusion in the last 6 months (aOR: 2.69;95% CI: 1.55–4.67) were independently found to be associated with a significantly higher risk of being hepatitis- HIV coinfected.

**Table 2:** Association of HIV and HBV/HCV infection

	HIV positive	HIV negative	OR (95% CI)	Fischer’s exact test
AgHBs				
Positive	26 (92.9%)	2 (7.1%)	14.1 (3.33–60.2)	< 0.001
Negative	271 (46.2%)	295 (53.8%)	1	
Anti-HCV Ab				
Positive	2 (100%)	0 (0.0)	5.03 (0.24–105.3)	0.297
Negative	295 (49.8%)	297 (50.2%)	1	

**Table 3:** Prevalence of HBV and HCV by age among HIV-positive and HIV-negative children

HIV-positive children	HBsAg Test			Anti-HCV Ab Test	
	Positive	Negative		Positive	Negative
Child’s age in months	n (%)	n (%)		n (%)	n (%)
< 60	5 (6.4)	73 (93.6)		0 (0.0)	78 (100)
60–120	9 (8.3)	100 (91.7)		1 (0.9)	108 (99.1)
> 120	12 (10.9)	98 (89.1)		1 (0.9)	109 (99.1)
**Total**	**26 (8.7)**	**271 (91.3)**		**2 (0.7)**	**295 (99.3)**
**HIV-negative children**	**HBsAg Test**			**Anti-HCV Ab Test**	
	**Positive**	**Negative**		**Positive**	**Negative**
Child’s age in months	n (%)	n (%)		n (%)	n (%)
< 60	0 (0.0)	199 (100)		0 (0.0)	199 (100)
60–120	1 (1.4)	71 (98.6)		0 (0.0)	72 (100)
> 120	1 (3.9)	25 (96.1)		0 (0.0)	26 (100)
**Total**	**2 (0.7)**	**295 (99.3)**		**0 (0.0)**	**297 (100)**

**Table 4:** Multifactorial analyses of HBV-HIV co-infection predictors by logistic regression

Medical history	HIV positive n (%)	HIV negative n (%)	OR (95% CI)	p-value	aOR (95% CI)	p-value
Have been hospitalized in the past 6 months						
Yes	65	79	0.77 (0.53–1.13)	0.180	10.7 (6.69–17.2)	0.000
No	232	247	1			
Have had surgery in the last 6 months						
Yes	12	14	0.85 (0.39–1.87)	0.688	2.95 (0.90–9.68)	0.074
No	285	283	1			
Have been transfused in the last 6 months						
Yes	32	17	1.99 (1.08–3.67)	0.025	4.94 (2.12–11.5)	0.000
No	265	309	1			
Have been tattooed in the last 6 months						
Yes	27	9	3.20 (1.47–6.92)	0.002	3.24 (1.18–8.92)	0.023
No	270	288	1			
Have been pierced in the past 6 months						
Yes	47	10	5.39(2.67–10.9)	0.000	4.26 (1.70–10.7)	0.002
No	250	287	1			
History of jaundice in the family environment						
Yes	33	18	1.93 (1.05–3.52)	0.028	1.91(0.66–5.54)	0.231
No	264	279	1			
History of jaundice in child						
Yes	14	7	2.04 (0.81–5.15)	0.120	2.69 (1.55–4.67)	0.000
No	283	290	1			

## Discussion

The main objective of this study was to determine the seroprevalence as well as risk factors of HBV and HCV co-infections in HIV- children. In particular, the study found that seroprevalence of HBV is higher in seropositive children than in seronegative.

HBV seroprevalence is higher than in all previous published data in HIV-negative children. Indeed, according to the latest estimates, DRC is among high intermediate endemic area (prevalence of HBsAg from 5 to 7%) for both children and adults [4, 12]. Our data also seem to show a significantly higher seroprevalence than those previously found in other subgroups of children, including 0% in poly-transfused children but 1.96% was found in HIV children in the Capital City of Kinshasa in the Western part of the DRC [21–23]. A community study carried out in the center of the country (Province of Maniema) noted a seroprevalence of 3.6% among children aged from 0 to 59 months [20].

It is well established that HBV transmission in Africa remains MTC and community-based during the first years of life [10, 19, 23, 24]. The possibility of sharing main routes of contamination as well as the importance of family context in transmission may be explained since our study noted that a history of jaundice was associated with a risk factor for HIV infection: OR 1.93 (95%CI: 1.05–3.52). p = 0.028.

The high prevalence of HBV in our children could be explained by the sharing of transmission ways with HIV but also by the fact that vaccination coverage is not yet optimal in all parts of the DRC [10, 20, 21, 23, 24]. This could explain the difference in seroprevalence with that of Province of Maniema [20]. Indeed, this last province is among the least covered by HBV vaccination. As for naive children, systematic vaccination remains the single most effective preventive factor in addition to systematic screening of the two viruses in pregnant women and administration of the first dose of the anti-HBV vaccine at birth.

Compared to HCV, although seroprevalence is slightly elevated in HIV + children but the difference is not statistically significant. In addition, it remains low compared to national estimates or to previous studies in other subgroups of children. Indeed, a prevalence of 13.5% was found among transfused children in Kinshasa and 2.8% in a community study in Maniema [20, 21]. Unfortunately, to our knowledge, there are no comparative studies on HIV + patients in the DRC. Nevertheless, other studies conducted in Africa have been able to find an association between HIV infections and HCV [25–27].

Apart from the small size of our sample, the risk factors for HCV contamination have already been pinpointed in previous studies, in particular case of blood transfusion and scarification [13]. The lack of difference in HCV seroprevalence between the 2 groups (HIV positive and naïve patients) observed in our study may be partly explained by the low frequency of risk factors in children compared to hepatitis B. Indeed, the risk of MTC transmission is 8–10 times less than during hepatitis B.

Nevertheless, it should be noted that the prevalence of co-infections with viral hepatitis across the country is not uniform and depends on several associated factors, such as cultural and religious factors as well as the quality of healthcare services. On the other hand, the cross-border movements of populations are likely to have transformed the epidemiology of these pathologies in the DRC, more particularly in the border provinces like South Kivu.

However, our study has some limitations that should be noted. Firstly, the limited size of our sample could not allow us to specify the importance of some considered factors. On the other hand, the fact that the HBV test was only performed during a single course could not help to confirm the possibility of chronic infection. In addition, the lack of use of PCR (Polymerase Chain Reaction) does not allow us to confirm the presence of an active infection.

In conclusion, our study showed that the prevalence of HBV in HIV-infected children is globally higher than in naive children. In contrast, the rate of HCV does not seem very different in the two groups likely due to low risk of MTC transmission with this virus. However, some factors seem to suggest a mutual influence of contamination routes in the family context. Systematic screening for these three viruses in pregnant women associated to a better therapeutic choice in co-infected children should remain the major recommendations. In addition, conventional preventive measures against hepatitis B, including blood transfusion safety, systematic vaccination as well as the increase of vaccination coverage, should continue to be promoted.

## Data Availability

The datasets used and analysed during the current study available from the corresponding author on reasonable request.

## Declarations

### Authors’ information

We would like to report the sudden death of our colleague, MSA, sometime after the completion of this study.
